# Supplementary material for: Reduced Graphene Oxide-Supported Iron-Cobalt Alloys as High-Performance Catalysts for Oxygen Reduction Reaction
Source: Nanomaterials (Basel). 2023 Oct 9;13(19):2735. doi: 10.3390/nano13192735 (PMC10574026; doi:10.3390/nano13192735)
Supplement: Supplementary file 1 [file nanomaterials-13-02735-s001.zip › nanomaterials-2583216-supplementary.pdf]

Supporting information

Preparation of graphene oxide

Graphene oxide solution was made by modified hummer's method, about 3g of scaled graphite powder was stirred with 120 mL of concentrated sulfuric acid to obtain a black viscous liquid, 12 g $KMnO_4$ was added under ice water bath conditions and stirred continuously for 15 h to oxidize it fully, and the final product was a dark green oily liquid. Pour the material into a beaker in an ice−water bath environment. Then the hydrogen peroxide was added drop by drop. It could be seen that a large number of bubbles appeared from the solution. Then the solution was continuously stirred until the bubbles disappeared. At last the brown liquid oxidation turns to be golden brown liquid. The remaining material in the reaction was washed away with 0.5 M sulfuric acid and ultrapure water until a golden paste was produced, which was graphene oxide. The obtained graphene oxide was poured into water and sonicated so that the graphene oxide was present as a monolayer. The obtained graphene oxide was configured as a dispersion solution with a concentration of 6 g/L.

Catalytic performance tests for ORR

The oxygen reduction reaction (ORR) catalytic performance of the prepared catalysts were tested in 0.1 M KOH solution. 5 mg of the sample were blended with 500 μL of a 1 wt % Nafion ethanol solution, and then sonicated until a homogeneous dispersion is formed. Next, 5μL of the obtained dispersion was pipetted onto a glassy carbon electrode with a diameter of 3 mm (0.6 mg/cm^2). For comparison, the Pt/C catalyst dispersion was prepared by the same method. Then, 5 μL of the Pt/C dispersion was also loaded onto a glassy carbon electrode. The catalyst dispersion was dried slowly in air until a uniform catalyst distribution across the electrode surface was obtained. Electrochemical measurements (cyclic voltammetry (CV), rotating disk electrode (RDE) and rotating ring-disk electrode (RRDE) measurements) were performed in a three-electrode cell system. A rotating glassy carbon disk and platinum ring electrode after loading with the electrocatalyst were used as working electrode, an Saturated calomel electrode (Hg/Hg_2Cl_2) as reference electrode, and a graphite

electrode as counter electrode. The electrochemical experiments were conducted in a 0.1 M KOH aqueous electrolyte (saturated with oxygen by bubbling O_2) for the oxygen reduction reaction. The potential range was cycled between -0.8 and 0.2 V at a scan rate of 5 mV/s at room temperature after purging O_2 gas for 30 min through the cell. In control experiments, CV measurements were performed in a Ar-saturated electrolyte. RDE measurements were scanned cathodically at a rate of 5 mV s^{-1} for different rotating speeds from 625 to 2025 rpm. All potential scale in this study are aligned to reversible hydrogen electrode (RHE) scale. The Nernst equation was used as the conversion formula for converting potentials from Ag/AgCl (3M KCl) to the RHE scale according to equation (1).

$$E_{RHE} = E_{Ag/AgCl} + 0.059pH + E^{\circ}_{Ag/AgCl} \qquad (1)$$

where $E_{Ag/AgCl}$ was the experimentally measured potential using Ag/AgCl as the reference electrode and $E^{\circ}_{Ag/AgCl}$ was 0.210 V.

The Koutecky−Levich (K−L) plots (J^{-1} vs $\omega^{-1/2}$) were analyzed at various electrode potentials. The slopes of the linear lines were used to calculate the number of electrons transferred (n) per oxygen molecule in the ORR process at the electrodes according to the K-L equation (2), and J_K can be calculated from the inverse of intercept

$$J=1/J_L+1/J_K=1/B\omega^{1/2}+1/J_K; \qquad (2)$$

$$B=0.62nFC_0(D_0)^{2/3}v^{-1/6}; \qquad (3)$$

$$J_K=nFkC_0 \qquad (4)$$

where J is the measured current density, J_K and J_L are the kinetic and diffusion limiting current densities, ω is the angular velocity of the rotating electrode (ω=2πN, N is the linear rotation speed), F is the Faraday constant (F = 96485 C mol^{-1}), C_0 is the concentration of O_2 in 0.1 M KOH (1.2×10^{-3} M), D^0 is the diffusion coefficient of O_2 in 0.1 M KOH (1.9×10^{-5} cm^2 s^{-1}), v is the kinematic viscosity of the electrolyte (0.01 cm^2) transfer rate constant. Rotating Ring-Disk Electrode (RRDE) Measurement: Catalyst inks and electrodes were prepared by the same method as above. The disk electrode was scanned at a rate of 5 mV s^{-1}, and the ring potential was constant at -0.07 V (vs. Hg/Hg_2Cl_2). Based on the RRDE results, the H_2O_2% and the electron transfer

number (n) could be calculated according to the following equation (5) and (6):

$$n=4\ I_{disk}/\ (I_{disk}+\ I_{ring}/N) \quad (5)$$

$$H_2O_2\ (\%) = (200I_{ring}/N)/\ (I_{disk} + I_{ring}/N) \quad (6)$$

where I_{disk} is disk current, I_{ring} is ring current, and N is current collection efficiency of the Pt ring. In this paper, the value of N is 0.39.

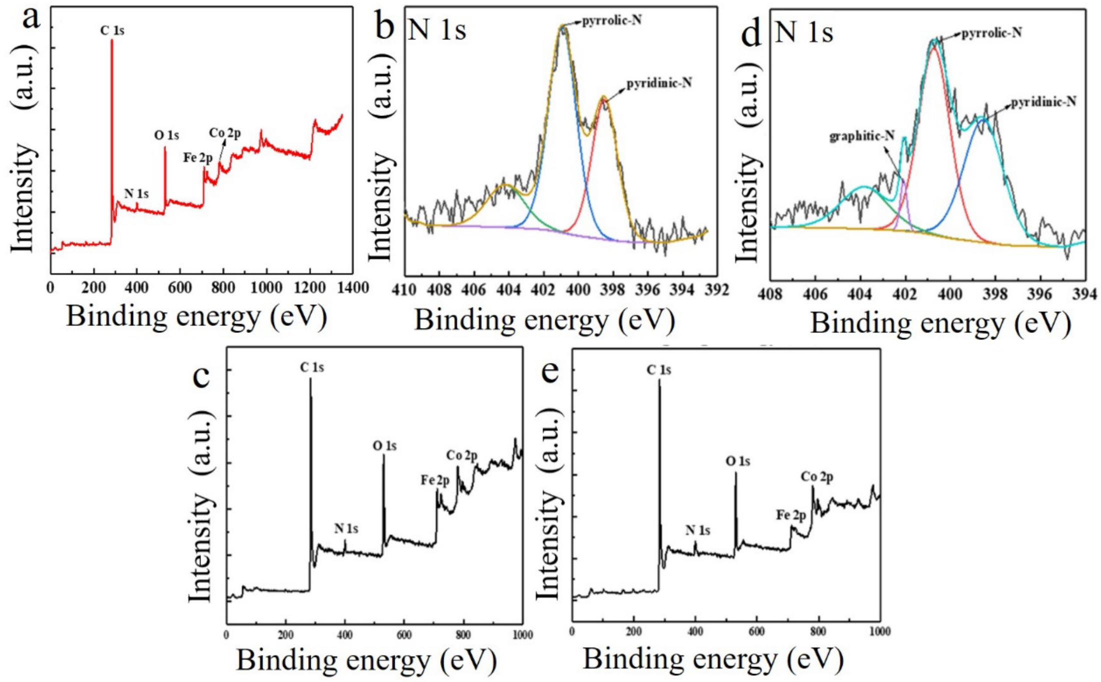

Figure S1. Full spectra XPS of FeCo@N−rGO−1 (a), High resolution N 1s (b) and Full spectra XPS (c) of FeCo@N−rGO−2; High resolution N 1s (d) and full spectra XPS (e) of FeCo@N−rGO−3.

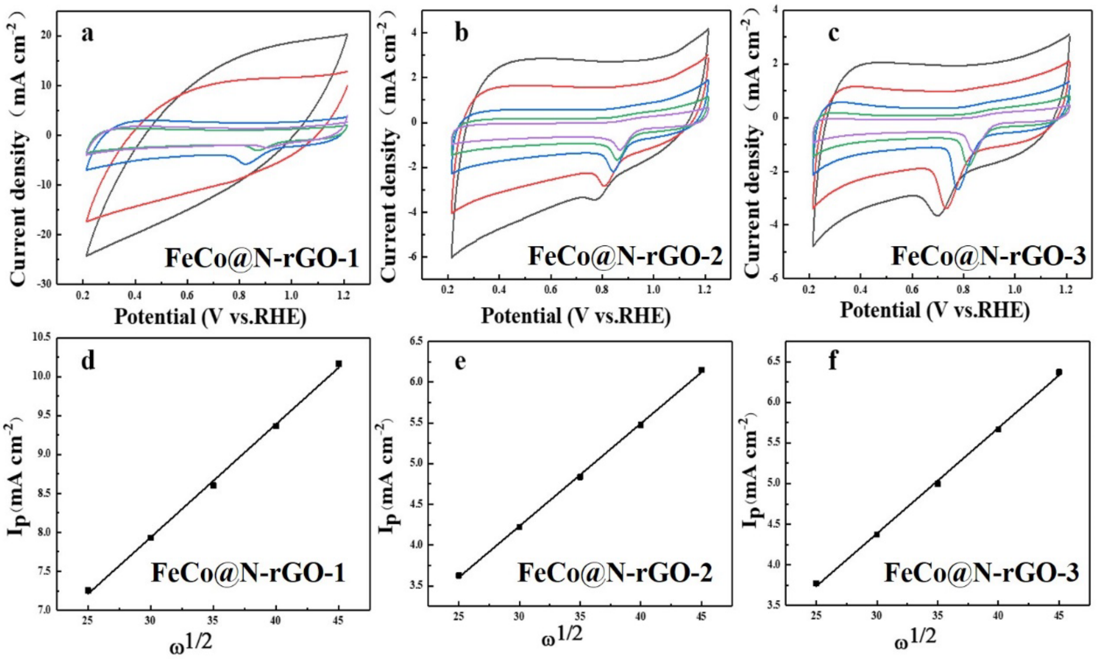

Figure S2. CV in different scan rate of FeCo@N−rGO−1 (a), FeCo@N−rGO−2 (b) and FeCo@N−rGO−3 (c). Ip−□□□□ of FeCo@N−rGO−1 (d), FeCo@N−rGO−2 (e) and FeCo@N−rGO−3 (f).

Table S1. The onset potential and half−wave potential of different materials

material	Onset potential (V vs. RHE)	Half-wave potential (V vs. RHE)
0.14Co0.01Fe–CB[1]	0.92	0.86
AA-Fe_2N@NC[2]	0.91	0.80
FeBNC−800[3]	0.968	0.838
CoNP@ FeNC−0.05[4]	0.93	0.85
Fe-Co_4N@N−C[5]	0.90	0.83

Table S2. $R_Ω$ and R_{ct} for FeCo@N−rGO−1, FeCo@N−rGO−2 and FeCo@N−rGO−3.

material	$R_Ω$（Ω）	R_{ct}（Ω）
FeCo@N-rGO-1	3.53	72.31
FeCo@N-rGO-2	5.00	69.04
FeCo@N-rGO-3	10.03	57.92

References

[1] W. Zhu, Y. Pei, J.C. Douglin, J. Zhang, H. Zhao, J. Xue, Q. Wang, R. Li, Y. Qin, Y. Yin, D.R. Dekel, M.D. Guiver, Multi-scale study on bifunctional Co/Fe–N–C cathode catalyst layers with high active site density for the oxygen reduction reaction, Applied Catalysis B: Environmental, 299 (2021) 120656.

[2] A. Zhu, L. Qiao, P. Tan, Y. Ma, W. Zeng, R. Dong, C. Ma, J. Pan, Iron-nitrogen-carbon species for oxygen electro-reduction and Zn-air battery: Surface engineering and experimental probe into active sites, Applied Catalysis B: Environmental, 254 (2019) 601-611.

[3] Q. Xu, H. Jiang, Y. Li, D. Liang, Y. Hu, C. Li, In-situ enriching active sites on co-doped Fe-Co_4N@N-C nanosheet array as air cathode for flexible rechargeable Zn-air batteries, Applied Catalysis B: Environmental, 256 (2019) 117893.

[4] K. Yuan, S. Sfaelou, M. Qiu, D. Lützenkirchen-Hecht, X. Zhuang, Y. Chen, C. Yuan, X. Feng, U. Scherf, Synergetic Contribution of Boron and Fe–Nx Species in Porous Carbons toward Efficient Electrocatalysts for Oxygen Reduction Reaction, ACS Energy Letters, 3 (2018) 252-260.

[5] J. Zhang, L. Dai, Heteroatom-Doped Graphitic Carbon Catalysts For Efficient Electrocatalysis Of Oxygen Reduction Reaction, ACS Catalysis, 5 (2015) 7244-7253.
